# Divergent Regulation of Mammary Lipogenesis by *trans*-10, *cis*-12 and *cis*-9, *trans*-11 CLA Isomers Is Determined by Receptor-Specific Signaling

**DOI:** 10.3390/ani15233418

**Published:** 2025-11-26

**Authors:** Siwen Cheng, Ang Zhao, Xueyan Lin, Zhonghua Wang

**Affiliations:** Key Laboratory of Efficient Utilization of Non-Grain Feed Resources (Co-Construction by Ministry and Province), Ministry of Agriculture and Rural Affairs, Shandong Provincial Key Laboratory of Animal Nutrition and Efficient Feeding, College of Animal Science and Technology, Shandong Agricultural University, Tai’an 271017, China

**Keywords:** conjugated linoleic acid, milk fat, G-protein-coupled receptor

## Abstract

Conjugated linoleic acid (CLA) isomers exhibit distinct biological activities in lipid metabolism, yet their roles in regulating mammary lipogenesis remain poorly understood. Understanding how individual CLA isomers influence lipid synthesis is essential for improving milk fat quality and developing targeted nutritional strategies. We assessed the effects of *trans*-10, *cis*-12 (t10c12-CLA) and *cis*-9, *trans*-11 (c9t11-CLA) isomers on lipid synthesis in goat mammary epithelial cells and investigated fatty acid receptor involvement. t10c12-CLA significantly suppressed key lipogenic proteins (FASN, ACACA, and SREBP-1) and impaired mitochondrial activity, as evidenced by reduced mitochondrial membrane potential, ATP/ADP ratio, and PGC-1α expression. Both isomers paradoxically increased lipid droplet accumulation; for t10c12-CLA, this phenomenon resulted from compensatory metabolic shifts due to weakened mitochondrial function. Critically, GPR40 inhibition reversed the antilipogenic effects and mitochondrial impairment caused by t10c12-CLA, establishing GPR40 as the key mediator. In contrast, c9t11-CLA had milder effects and preserved mitochondrial activity, with no definitive receptor dependence established. Transcriptomic analysis confirmed isomer-specific gene expression patterns, with t10c12-CLA broadly affecting lipid metabolism and mitochondrial pathways in a GPR40-dependent manner. These findings reveal that the divergent effects of CLA isomers are dictated by differential receptor engagement, providing insights for precision nutritional strategies in dairy production.

## Simple Summary

Milk fat is an important part of dairy nutrition and economics, but its production can be reduced by certain dietary components. This study examined how two similar fatty acid molecules called CLA isomers affect fat-making processes in mammary gland cells from goats. Although these isomers have nearly identical structures, they produce very different effects. One isomer (t10c12-CLA) strongly suppressed the production of proteins needed to make milk fat and damaged cellular energy factories called mitochondria, while the other isomer (c9t11-CLA) had much milder effects and preserved normal cellular function. By investigating the underlying mechanisms, researchers discovered that t10c12-CLA works through a specific cellular receptor called GPR40 to trigger these dramatic changes, whereas c9t11-CLA does not strongly activate this same pathway. Importantly, blocking GPR40 reversed most of the damaging effects of t10c12-CLA. These findings explain why structurally similar molecules can have dramatically different biological effects and suggest new strategies for controlling milk composition through targeted nutritional approaches in dairy farming.

## 1. Introduction

Milk fat is a critical determinant of both the economic and nutritional value of dairy products, providing essential fatty acids and bioactive lipids important for human health [1,2]. The synthesis of milk fat occurs within mammary epithelial cells (MECs) through an intricate network of metabolic and signaling pathways [3,4,5]. Understanding the molecular mechanisms underlying this process is essential for improving milk composition to meet consumer health expectations and production efficiency goals. Among the dietary factors influencing milk fat synthesis, conjugated linoleic acids (CLAs) have emerged as potent naturally occurring modulators with unique biological properties.

The two major CLA isomers—*cis*-9, *trans*-11 (c9t11-CLA) and *trans*-10, *cis*-12 (t10c12-CLA)—present intriguing biological contrasts despite their structural similarity [6]. While t10c12-CLA robustly inhibits lipogenesis and contributes to milk fat depression in ruminants [7,8], c9t11-CLA, the more abundant isomer in dairy products, lacks this inhibitory effect and is instead associated with anti-inflammatory and metabolic benefits [9,10]. This functional divergence strongly implies the existence of isomer-specific molecular recognition and signaling mechanisms. However, the molecular basis for this differential recognition has remained elusive, representing a critical knowledge gap in our understanding of CLA biology. In cattle, t10c12-CLA consistently suppresses milk fat synthesis by 25–50% [11], whereas in rodent adipocytes, it reduces triglyceride accumulation [12]. However, paradoxical increases in intracellular lipid storage have been reported under specific metabolic contexts [13], suggesting context-dependent effects that remain mechanistically unexplained.

A critical unresolved paradox is that t10c12-CLA inhibits lipogenic gene expression yet some studies report unchanged or increased lipid droplet formation in vitro [14,15,16,17]. This discrepancy may reflect compensatory metabolic shifts. Previous studies have extensively characterized the downstream effects of CLA isomers, including t10c12-CLA-mediated suppression of key lipogenic enzymes such as fatty acid synthase (FASN) and acetyl-CoA carboxylase alpha (ACACA) [18] and transcription factors such as SREBP-1 [19,20,21]. However, the upstream signaling events that trigger these changes remain poorly defined. Recent studies have identified fatty acid-sensing G protein-coupled receptors (GPCRs), particularly GPR40 and GPR120, as crucial metabolic regulators [22,23,24]. These receptors bind long-chain fatty acids and influence lipid metabolism, inflammation, and cellular homeostasis [25,26]. Whether the structural differences between CLA isomers result in differential receptor engagement and consequently distinct downstream signaling effects remains unexplored in mammary tissue.

Critically, GPCR activation can initiate downstream signaling cascades that modulate mitochondrial function [27,28]. Mitochondria supply both ATP and biosynthetic intermediates required for fatty acid synthesis, whose functions are regulated by factors such as PGC-1α [29,30,31]. Given the divergent metabolic phenotypes induced by t10c12-CLA versus c9t11-CLA, isomer-specific GPCR signaling may lead to different effects on mitochondrial activity. Such mitochondrial alterations could significantly contribute to the contrasting effects of the two isomers on lipogenesis. However, the specific mitochondrial responses triggered by CLA isomers via GPCR pathways and how these differences mechanistically underlie their distinct metabolic activities remain unclear.

Therefore, the present study was designed to elucidate the molecular mechanisms underlying the divergent effects of CLA isomers on mammary lipogenesis. We hypothesize that t10c12-CLA and c9t11-CLA exert differential regulatory effects on lipid synthesis in goat mammary epithelial cells (GMECs) through distinct receptor-mediated signaling pathways, specifically involving GPR40 and GPR120.

The specific objectives of this study were to (1) compare the effects of t10c12-CLA and c9t11-CLA on lipid droplet formation and key lipogenic gene expression in GMECs; (2) identify the receptor-specific signaling pathways mediating these isomer-specific effects via pharmacological modulators; (3) characterize the transcriptomic profiles induced by each CLA isomer to reveal comprehensive molecular mechanisms; and (4) assess the impact of CLA treatment on cellular energy metabolism and mitochondrial function. This research addresses a critical knowledge gap in understanding the mechanistic basis for CLA isomer-specific effects on mammary lipogenesis, providing essential insights for precision nutrition strategies in dairy production and advancing our understanding of bioactive fatty acid regulation of cellular metabolism.

## 2. Materials and Methods

### 2.1. Primary Cell Isolation and Culture

Primary goat mammary epithelial cells (GMECs) were isolated from lactating Laoshan dairy goats at peak lactation as described previously [32]. The use of animal tissues was approved by the Institutional Animal Use Committee of Shandong Agricultural University. The cells were cultured in complete medium [Dulbecco’s modified Eagle’s medium (DMEM)/F12 medium (C11330500BT; Gibco, Grand Island, NY, USA) supplemented with 5 μg/mL transferrin (T8010; Solarbio, Beijing, China), 5 μg/mL insulin (I8040; Solarbio), 1 μg/mL hydrocortisone (IH0100; Solarbio), 10 ng/mL epidermal growth factor (P00033; Solarbio), 1% (*w*/*v*) penicillin–streptomycin (15140122; Gibco), and 10% (*v*/*v*) fetal bovine serum (FBS) (10099141; Gibco)] in a humidified atmosphere containing 5% (*v*/*v*) CO_2_. Cells at passages 3–5 were used in all experiments to ensure viability and consistency. GMECs were chosen because they closely mimic the lipid metabolic activity observed in vivo, making them an ideal model for studying conjugated linoleic acid (CLA)-induced effects on milk fat synthesis. Cells were used at 60% confluence in their undifferentiated proliferative state to maintain metabolic plasticity

### 2.2. Cell Treatments and Experimental Design

GMECs were seeded in 6-well plates at 2 × 10^5^ cells per well and cultured to ~60% confluence to maintain active proliferation and avoid contact inhibition. CLA isomers were purchased at ≥98% purity (verified by GC-MS, certificates provided by manufacturer). Stock solutions of *cis*-9, *trans*-11 CLA (c9t11-CLA) and *trans*-10, *cis*-12 CLA (t10c12-CLA) (MedChemExpress, Monmouth Junction, NJ, USA) were prepared in dimethyl sulfoxide (DMSO) at 200 mM and stored at −20 °C. The working solutions (100 μM) were freshly prepared in culture medium. For all experiments, c9,t11-CLA and t10,c12-CLA were used at 100 μM, a dose selected based on previous studies in bovine/caprine mammary epithelial cells showing clear biological effects of CLA isomers in vitro [9,33]. Vehicle controls received ≤0.05% (*v*/*v*) DMSO. The GPR40 and GPR120 modulators (GW-1100, fasiglifam, AH-7614, and TUG-891; 100 μM) were purchased from MedChemExpress (Monmouth Junction, NJ, USA). The concentrations were chosen according to the manufacturer’s instructions and fall within the recommended working ranges for cell-based assays. At these concentrations, no obvious cytotoxicity or morphological changes were observed during the 24 h treatments.

All the treatments were performed for 24 h in three series. Series 1 assessed direct CLA effects via vehicle control (0.05% DMSO), 100 μM c9t11-CLA, and 100 μM t10c12-CLA. Series 2 examined GPR40-mediated mechanisms by pretreating cells with 100 μM GPR40 antagonist GW-1100 or 100 μM GPR40 agonist fasiglifam (MedChemExpress) for 1 h before the addition of CLA, including modulator-alone and combination treatments. Series 3 used an identical design with GPR120-specific modulators: 100 μM AH-7614, a GPR120 antagonist, and 100 μM TUG-891, a GPR120 agonist (MedChemExpress). All experiments were conducted via three independent biological replicates, with each replicate representing a complete and separate experimental run from cell seeding to harvest and analysis.

### 2.3. Protein Electrophoresis and Immunoblotting

GMECs were resuspended on ice in lysis buffer (AIWB-012; Affinibody LifeScience, Stockholm, Sweden) and vortexed thoroughly. Lysates were clarified by centrifugation (10 min at 14,000× *g*, 4 °C) and quantified with a BCA Rapid Protein Assay Kit (P0398L; Beyotime, Shanghai, China). The samples were incubated at 70 °C for 10 min in Laemmli sample buffer, separated by SDS–PAGE, and transferred to polyvinylidene fluoride (PVDF) membranes (ISEQ00010; Sigma-Aldrich, St. Louis, MO, USA). The membranes were blocked with StartingBlock Blocking Buffer (37579; Thermo Scientific, Waltham, MA, USA) for 1 h and then incubated with primary and secondary antibodies diluted in Immunoreaction Solution (NKB-101; Toyobo, Osaka, Japan).

The primary antibodies used (species, vendor, catalog number, dilution) were FASN (rabbit, Proteintech, Rosemont, IL, USA, cat# 10624-2-AP; 1:2500), SREBP-1 (rabbit, Proteintech cat# 82684-3-RR; 1:2500), PGC-1α (mouse, Proteintech cat# 66369-1-Ig; 1:2500), ACACA (rabbit, Proteintech cat# 21923-1-AP; 1:2500), α-Tubulin (rabbit, Cell Signaling Technology, Danvers, MA, USA, cat# 2125S; 1:5000), Vinculin (rabbit, Abcam, Cambridge, UK, cat# ab129002; 1:5000), and β-actin (rabbit, Proteintech cat# 81115-1-RR; 1:10,000). The secondary antibodies used (species, vendor, catalog number, dilution) were as follows: HRP–goat anti-rabbit recombinant secondary antibody (goat, Proteintech cat# RGAR001; 1:10,000) and HRP–goat anti-mouse recombinant secondary antibody (goat, Proteintech cat# RGAM001; 1:10,000).

Chemiluminescent images were captured with a Vilber imaging system (Vilber Lourmat, Marne-la-Vallée, France), analyzed with ImageJ software (version 1.54f, National Institutes of Health, Bethesda, MD, USA), and prepared for presentation in Adobe Photoshop (version 2024) and Illustrator (version 2024) (Adobe Systems Inc., San Jose, CA, USA).

### 2.4. RNA Sequencing and Analysis

RNA sequencing was performed on three independent biological replicates per treatment group (*n* = 3), with each replicate representing cells from a separate animal and experimental run. No technical replicates were pooled for library preparation. Total RNA was isolated via TRIzol reagent (Invitrogen, Carlsbad, CA, USA) according to the manufacturer’s instructions. RNA integrity was assessed by 1% agarose gel electrophoresis; purity and concentration were measured with a NanoDrop 2000 spectrophotometer (Thermo Scientific). The RNA integrity number (RIN) was determined via an Agilent DNF-471 RNA Kit on an Agilent 5300 system (Agilent Technologies, Santa Clara, CA, USA).

The sequencing libraries were prepared via the Illumina Stranded mRNA Prep Kit (Illumina, San Diego, CA, USA), ligated, and sequenced on the Illumina NovaSeq X Plus platform. The raw data were quality filtered with fastp (version 0.23.2) [34], and the clean reads were aligned to the reference genome via HISAT2 (version 2.2.1) [35]. Transcript abundances were quantified with RSEM (version 1.3.3) [36], and differential expression analysis was performed with DESeq2 (version 1.40.2) [37]; false discovery rate (FDR) < 0.05, |log_2_-fold change| ≥ 1). Gene Ontology (GO) annotation included biological process, cellular component, and molecular function categories via GOATOOLS (version 1.4.5) [38] with Fisher’s exact test and multiple-comparison correction (Bonferroni, Holm, Šidák, FDR); terms with FDR-adjusted *p* < 0.05 were considered significant. Kyoto Encyclopedia of Genes and Genomes (KEGG) pathway enrichment was analyzed with Fisher’s exact test and Benjamini–Hochberg correction (*p* < 0.05).

### 2.5. Lipid Droplet Staining

GMECs were grown on #1.5 coverslips in 12-well plates. After treatment, the cells were fixed with 3% paraformaldehyde at room temperature for 20 min, washed three times with phosphate-buffered saline (PBS), and blocked in 2% bovine serum albumin (BSA) in PBS containing 0.2% Triton X-100 for 30 min. Coverslips were incubated in the dark with 2 μM BODIPY 493/503 (Invitrogen) prepared in 1% BSA/PBS with 0.1% Triton X-100 for 1 h. Nuclei were stained with Hoechst 33342 (Invitrogen), rinsed, and mounted on slides. Images were collected via a Nikon Eclipse Ts2R microscope (Nikon, Tokyo, Japan) with 405 nm or 488 nm excitation lines and analyzed via FIJI (ImageJ; https://imagej.net/ij/, accessed on 15 May 2024). The lipid droplet size and number per cell were quantified via the Aggrecount plugin (https://aggrecount.github.io/, accessed on 15 May 2024).

### 2.6. Mitochondrial Membrane Potential and ATP Production Analyses

The mitochondrial membrane potential (MMP) was measured via JC-1 staining (C2003S; Beyotime) according to the manufacturer’s instructions, and the ATP/ADP ratio was determined with an ADP/ATP ratio assay kit (MAK135; Sigma-Aldrich) according to the manufacturer’s protocol.

### 2.7. Statistical Analysis

All experiments were performed with three independent biological replicates, and each biological replicate included three technical replicates. The data are expressed as the means ± standard errors of the means (SEMs). Statistical analyses were conducted in GraphPad Prism (version 10.2.2; GraphPad Software, San Diego, CA, USA). Differences among multiple groups were analyzed by one-way ANOVA followed by Tukey’s multiple comparison test; pairwise comparisons were performed with Student’s *t* test. Statistical significance was set at *p* < 0.05. For transcriptome analyses, differential gene expression was considered significant at FDR < 0.05 and |log_2_-fold change| ≥ 1; pathway enrichment was considered significant at Benjamini–Hochberg adjusted *p* < 0.05.

## 3. Results

### 3.1. The t10,c12-CLA Isomer Selectively Attenuates Lipogenic Protein Expression in Mammary Epithelial Cells

**Figure 1:**
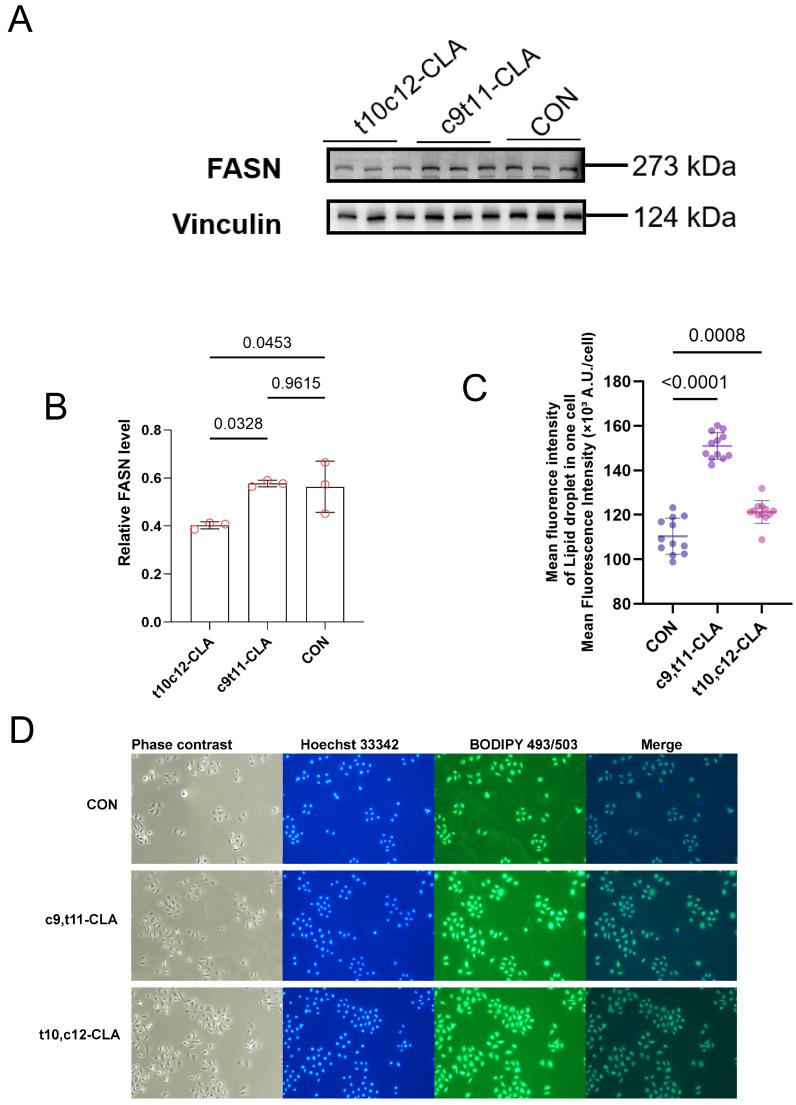
Effect of CLA Isomers on FASN Expression and Lipid Droplet Formation. Primary undifferentiated GMECs at ~60% confluence were treated with vehicle control (CON, 0.05% DMSO), 100 μM c9,t11-CLA, or 100 μM t10,c12-CLA for 24 h to assess impacts on fatty acid synthase (FASN) expression and lipid droplet accumulation. (**A**) Representative Western blot images showing FASN (273 kDa) and Vinculin (124 kDa) as loading control. (**B**) Quantification of the Western blot in A. (**C**) Quantification of lipid droplet fluorescence intensity shown in (**D**), expressed as mean fluorescence per cell (*n* = 100–150 cells per group from three independent experiments). (**D**) Representative fluorescence microscopy images of lipid droplets stained with Bodipy 493/503 (green); nuclei counterstained with DAPI (blue). Scale bar = 200 μm. Increased lipid droplet accumulation is evident, particularly with t10,c12-CLA. Data are means ± SEM; statistical significance determined by one-way ANOVA with Tukey’s post hoc test.

To test the central hypothesis that mammary epithelial cells can distinguish between structurally similar CLA isomers, we compared the effects of c9,t11-CLA and t10,c12-CLA on the abundance of key lipogenic proteins. Undifferentiated primary GMECs at ~60% confluence were exposed to 100 μM c9,t11-CLA or t10,c12-CLA for 24 h, after which protein levels were quantified via Western blot analysis. The t10,c12-CLA isomer markedly decreased the expression of fatty acid synthase (FASN), a rate-limiting enzyme in de novo lipogenesis (Figure 1A,B). FASN protein abundance was reduced to 42 ± 8% of the control level (*p* = 0.0453), whereas c9,t11-CLA caused no measurable change (98 ± 12% of control, *p* = 0.9615). This difference was statistically significant.

These findings indicate that, despite their structural similarity, only t10,c12-CLA has a significant antilipogenic effect on mammary epithelial cells, suggesting the existence of isomer-specific recognition mechanisms.

### 3.2. Both CLA Isomers Increase Lipid Droplet Accumulation Despite Their Divergent Effects on Lipogenic Enzyme Abundance

After confirming that t10,c12-CLA suppresses lipogenic protein expression, we assessed intracellular lipid content. Representative images (Figure 1D) and quantification (Figure 1C) showed that both CLA isomers. Neutral lipid accumulation was visualized via BODIPY 493/503 staining, followed by fluorescence microscopy and quantitative image analysis.

In contrast to expectations, both CLA isomers increased lipid droplet formation compared with the vehicle control (Figure 1C). T10,c12-CLA increased the number of lipid droplets per cell by 2.8 ± 0.4-fold (*p* = 0.0008) and the total droplet area by 3.2 ± 0.5-fold (*p* = 0.0004). Similarly, c9,t11-CLA increased the droplet number by 2.3 ± 0.3-fold (*p* = 0.0021) and the droplet area by 2.6 ± 0.4-fold (*p* = 0.0017). No statistically significant differences in lipid accumulation were observed between isomers (*p* = 0.34 for droplet number; *p* = 0.42 for droplet area).

These unexpected increases in lipid storage, despite the suppression of lipogenic enzymes by t10,c12-CLA, suggest that CLA isomers influence cellular lipid homeostasis through mechanisms beyond de novo synthesis, potentially involving changes in fatty acid uptake, oxidation, or catabolism.

### 3.3. The t10,c12-CLA Isomer Selectively Compromises Mitochondrial Integrity and Energy Balance

To address the paradox of increased lipid accumulation despite reduced lipogenic capacity, we examined the effects of CLA isomers on mitochondrial function and cellular energetics. The mitochondrial membrane potential was assessed by JC-1 staining, and the cellular ATP levels were measured.

**Figure 2:**
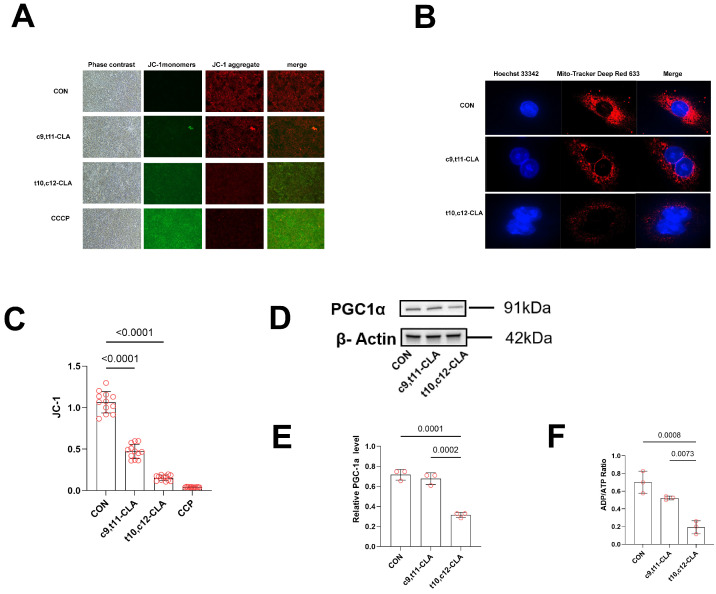
Mitochondrial Function and Dynamics with CLA Variants. Cells were treated with control, c9,t11-CLA, t10,c12-CLA, or CCCP (mitochondrial uncoupler) to evaluate mitochondrial membrane potential, morphology, biogenesis, and energy status. (**A**) Representative images of JC-1 staining: phase contrast, JC-1 monomers (green), and JC-1 aggregates (red) showing mitochondrial membrane potential; CCCP induces marked depolarization. (**B**) Mitochondrial morphology assessed by Mito-Tracker Deep Red 633 (red) staining, with Hoechst 33342 (blue) for nuclei and merged views; treatments alter mitochondrial distribution and fragmentation. (**C**) Quantification of JC-1 monomer-to-aggregate ratio, with significant increases indicating dysfunction (*n* = 3). (**D**) Representative Western blot of PGC-1α (91 kDa) and α-tubulin (55 kDa) as loading control. (**E**) Quantification of the Western blot in D. (**F**) ATP/ADP ratio, with significant decreases reflecting energy impairment (*n* = 3). Data are means ± SEM; statistical significance by one-way ANOVA with Tukey’s post hoc test.

Compared with a milder decrease induced by c9,t11-CLA (84 ± 9%, *p* = 0.031), exposure to t10,c12-CLA substantially reduced the mitochondrial membrane potential to 58 ± 7% of that of the control (*p* < 0.001) (Figure 2A,C). Confocal microscopy revealed extensive mitochondrial fragmentation and perinuclear clustering in t10,c12-CLA-treated cells, whereas c9,t11-CLA preserved relatively intact mitochondrial morphology (Figure 2B).

Consistent with these changes, t10,c12-CLA reduced the expression of PGC-1α, a key regulator of mitochondrial biogenesis, to 35 ± 8% of that of the control (*p* = 0.0007), whereas c9,t11-CLA had no significant effect (92 ± 11%, *p* = 0.42) (Figure 2D,E). Moreover, t10,c12-CLA decreased the cellular ATP/ADP ratio by 65% (*p* = 0.0003), indicating marked energy depletion, whereas c9,t11-CLA had no measurable effect (*p* = 0.37) (Figure 2F).

These results suggest that t10,c12-CLA selectively disrupts mitochondrial function and energy homeostasis, possibly triggering compensatory lipid storage as cells shift from oxidative phosphorylation toward lipid accumulation.

### 3.4. GPR40, but Not GPR120, Mediates the Antilipogenic Effects of t10,c12-CLA

We next investigated whether two known long-chain fatty acid-sensing G protein-coupled receptors, GPR40 and GPR120, mediate the observed isomer-specific effects. Specific receptor agonists and antagonists were used.

**Figure 3:**
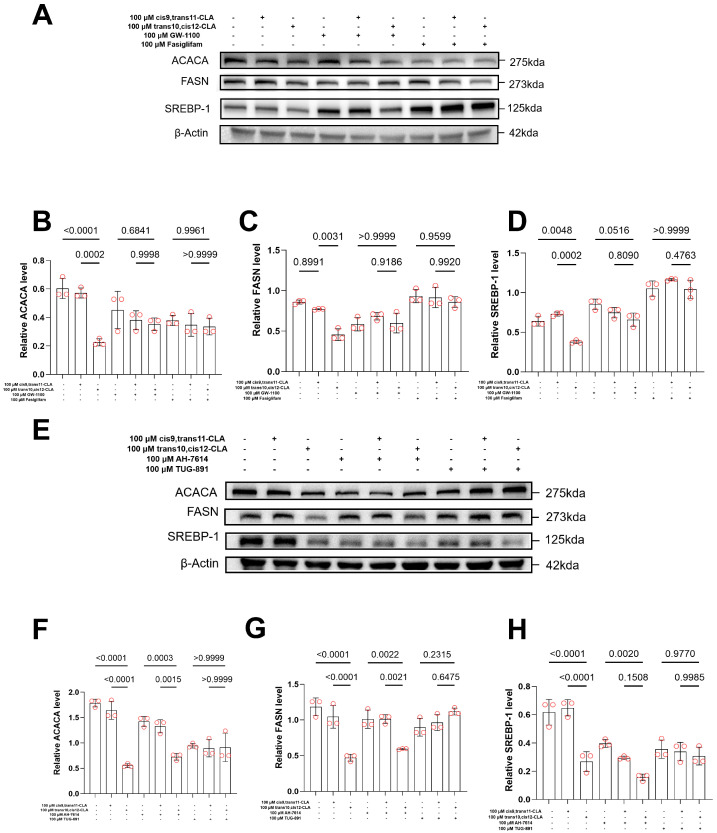
Effects of CLA Isomers and Modulators on Lipogenic Protein Expression. Goat mammary epithelial cells (GMECs) were pretreated with GPR40 or GPR120 modulators—including respective antagonists and agonists (activators)—to assess impacts on acetyl-CoA carboxylase (ACACA), fatty acid synthase (FASN), and sterol regulatory element-binding protein 1 (SREBP1) expression. (**A**–**D**) GPR40 modulation: Pretreatment with GPR40 antagonist GW-1100 (100 μM) fully reversed trans-10,cis-12-CLA (t10,c12-CLA)-induced suppression of ACACA, FASN, and SREBP1, while GPR40 agonist/activator fasiglifam (100 μM) mimicked t10,c12-CLA effects. Representative Western blots are shown in (**A**), with quantifications normalized to β-actin in (**B**–**D**). (**E**–**H**) GPR120 modulation: Neither t10,c12-CLA nor cis-9,trans-11-CLA (c9,t11-CLA) effects were blocked by GPR120 antagonist AH-7614 (100 μM), though GPR120 agonist/activator TUG-891 (100 μM) independently suppressed all proteins. Representative Western blots are shown in (**E**), with quantifications normalized to β-actin in (**F**–**H**). Data are means ± SEM (*n* = 3 independent experiments); significance determined by one-way ANOVA with Tukey’s post hoc test.

Western blot analysis confirmed the stronger inhibitory effect of t10,c12-CLA than of c9,t11-CLA on lipogenic proteins (ACACA, FASN, and SREBP1) (Figure 3A–H). For example, t10,c12-CLA reduced ACACA expression to 0.25 ± 0.03-fold that of the control (*p* < 0.001), whereas c9,t11-CLA reduced ACACA expression to 0.55 ± 0.08-fold (*p* < 0.05). Similar patterns were observed for FASN (t10,c12: 0.58 ± 0.07-fold, *p* < 0.01; c9,t11: 0.75 ± 0.06-fold, *p* < 0.05) and SREBP1 (t10,c12: 0.50 ± 0.12-fold, *p* < 0.01; c9,t11: 0.70 ± 0.05-fold, *p* < 0.05).

When GPR40 was blocked with GW-1100 (Figure 3A–D), the suppression of lipogenic proteins by t10,c12-CLA was significantly reversed: ACACA, 1.42 ± 0.08-fold (*p* < 0.01); FASN, 1.68 ± 0.15-fold (*p* < 0.001); SREBP1, 1.75 ± 0.18-fold (*p* < 0.05) compared with t10,c12-CLA alone. GPR40 activation with fasiglifam moderately inhibited ACACA (*p* < 0.05), supporting its role in lipogenesis regulation.

In contrast, antagonism of GPR120 with AH-7614 (Figure 3E–H) had minimal effects on lipogenic protein levels (*p* > 0.05). Direct GPR120 agonism with TUG-891 strongly reduced ACACA (0.32 ± 0.04-fold, *p* < 0.01), FASN (0.45 ± 0.08-fold, *p* < 0.001), and SREBP1 (0.28 ± 0.06-fold, *p* < 0.001), indicating that GPR120 activation can inhibit mammary lipogenesis independently of t10,c12-CLA.

Taken together, these results indicate that t10,c12-CLA primarily exerts antilipogenic effects via GPR40, whereas GPR120 has an inhibitory effect when directly activated but is not involved in t10,c12-CLA’s mechanism.

### 3.5. Transcriptomic Profiling Supports Isomer Specific, Receptor-Dependent Regulation

**Figure 4:**
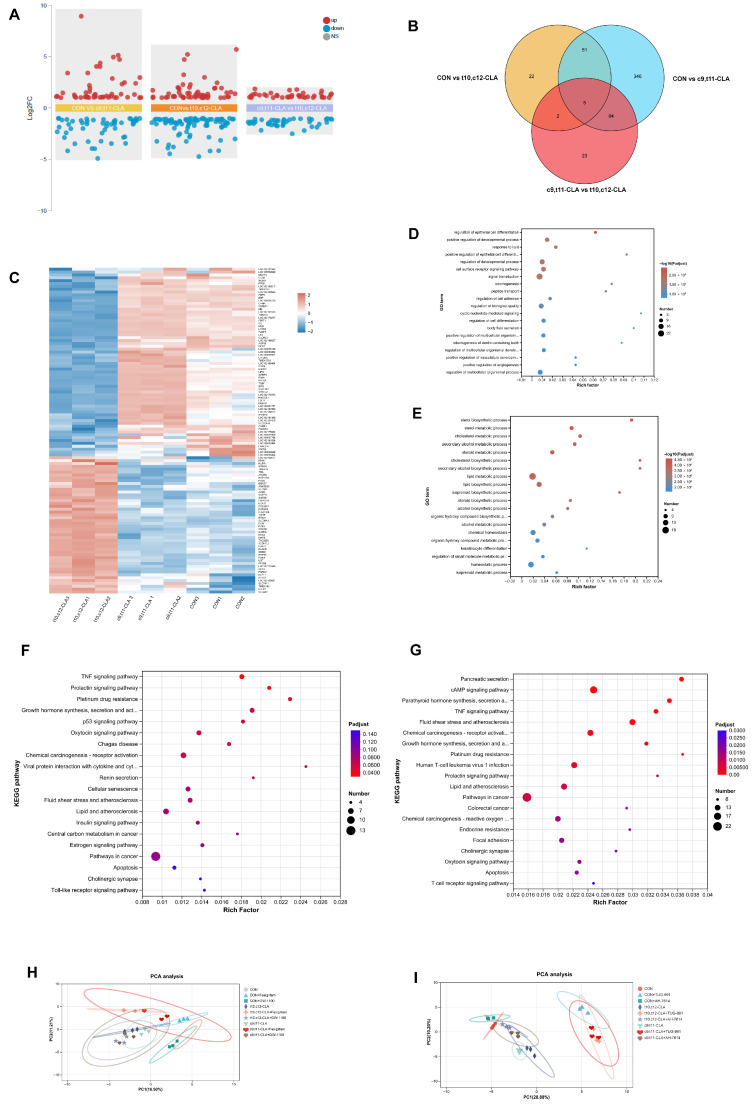
Transcriptomic profiling substantiates isomer-specific and receptor-contingent regulation. Transcriptomic profiling elucidates the molecular underpinnings of the divergent metabolic influences exerted by conjugated linoleic acid (CLA) isomers on goat mammary epithelial cells (GMECs). Cells were subjected to 24 h exposures to c9,t11-CLA, t10,c12-CLA, or selective agonists/antagonists targeting GPR40 or GPR120, succeeded by RNA sequencing. (**A**) Volcano plots delineating differentially expressed genes (DEGs) across pairwise contrasts: control (CON) versus c9,t11-CLA, CON versus t10,c12-CLA, and t10,c12-CLA versus c9,t11-CLA. (**B**) Venn diagram portraying the intersections of DEGs among the aforementioned contrasts (CON versus c9,t11-CLA, CON versus t10,c12-CLA, and t10,c12-CLA versus c9,t11-CLA). (**C**) Hierarchical clustering of gene expression signatures spanning the CON, c9,t11-CLA, and t10,c12-CLA cohorts. (**D**) Gene Ontology (GO) term enrichment for DEGs identified in the CON versus c9,t11-CLA comparison. (**E**) GO term enrichment for DEGs in the CON versus t10,c12-CLA comparison. (**F**) Kyoto Encyclopedia of Genes and Genomes (KEGG) pathway enrichment for DEGs in the CON versus c9,t11-CLA comparison. (**G**) KEGG pathway enrichment for DEGs in the CON versus t10,c12-CLA comparison. (**H**) Principal component analysis (PCA) encompassing treatments with GPR40 agonists and antagonists. (**I**) PCA encompassing treatments with GPR120 agonists and antagonists.

To explore the molecular basis of the differential effects of CLA isomers, undifferentiated GMECs were treated with c9,t11-CLA, t10,c12-CLA, or vehicle for 24 h and subjected to RNA-seq analysis. Compared with the control treatment, t10,c12-CLA altered the expression of 108 genes (42 upregulated, 66 downregulated), whereas c9,t11-CLA altered 80 genes (38 upregulated, 42 downregulated) (|log_2_FC| ≥ 2, FDR < 0.05) (Figure 4A). T10,c12-CLA had a broader transcriptomic impact, which was consistent with its stronger physiological effects. Venn analysis revealed 56 genes regulated by both isomers, with 44 unique to t10,c12-CLA and 43 unique to c9,t11-CLA (Figure 4B). Hierarchical clustering of the top 200 variable genes grouped the samples by treatment, with t10,c12-CLA forming the most distinct cluster from the control (Figure 4C).

Gene Ontology analysis revealed that t10,c12-CLA significantly downregulated “lipid biosynthetic process” (GO:0008610, *p* = 1.25 × 10^−7^) and “lipid metabolic process” (GO:0006629, *p* = 1.03 × 10^−7^) (Figure 4E), whereas c9,t11-CLA caused weaker changes in these pathways (Figure 4D). KEGG pathway analysis confirmed that t10,c12-CLA suppressed lipid metabolism pathways (Figure 4G), whereas c9,t11-CLA aligned with immune-related pathways (Figure 4F).

Principal component analysis of the results of the receptor modulation experiments revealed that the samples from the GPR40 antagonist-treated group clustered closer to the control samples than those from the CLA-only treatment groups (Figure 4H). This pattern was only modestly affected by GPR120 modulation (Figure 4I), underscoring the primary role of GPR40 in mediating the transcriptomic effects of t10,c12-CLA.

Overall, these data provide molecular evidence that CLA isomers elicit distinct metabolic effects in mammary epithelial cells through receptor-specific signaling: t10,c12-CLA predominantly engages GPR40 to repress lipogenesis and impair mitochondrial function, whereas c9,t11-CLA exerts more moderate effects linked to GPR120 activation.

## 4. Discussion

Despite their structural similarity, the CLA isomers c9,t11 and t10,c12 have markedly different effects on milk fat synthesis, with t10,c12-CLA causing milk fat depression and c9,t11-CLA lacking this inhibitory activity. To date, the molecular mechanisms underlying this isomer-specific regulation have remained elusive, limiting our ability to predict and manipulate their metabolic effects. This study provides the first comprehensive mechanistic explanation for how mammary epithelial cells distinguish between these isomers and achieve different metabolic responses through the application of these two CLAs.

Our findings demonstrate that t10,c12-CLA, but not c9,t11-CLA, notably suppresses key lipogenic proteins (FASN, ACACA, and SREBP-1) and severely impairs mitochondrial function through a GPR40-dependent mechanism. Although previous studies have documented the antilipogenic properties of t10,c12-CLA in mammary tissues [4,9,33,39,40] our work provides evidence linking these effects to the activation of a specific fatty acid receptor in mammary epithelial cells, thereby resolving a critical mechanistic gap in understanding CLA-mediated milk fat depression.

Unexpectedly, the application of both isomers increased lipid droplet accumulation, indicating that t10,c12-CLA may induce a fundamental metabolic shift from fatty acid oxidation to storage. Transcriptomic profiling confirmed these distinct biological processes, with t10,c12-CLA regulating the expression of 108 genes versus only 80 genes for c9,t11-CLA. Importantly, GPR40 antagonism abolished most of the effects of t10,c12-CLA, strongly confirming that GPR40 is the critical molecular discriminator that enables isomer-specific responses.

### 4.1. Receptor Specificity Determines Isomer Recognition

Previous studies have documented the downstream effects of CLA isomers without identifying the initial recognition mechanism. Our identification of GPR40 as the primary mediator of the effects of t10,c12-CLA in mammary epithelial cells is strongly supported by the substantial reversal of the antilipogenic effects of t10,c12-CLA via GPR40 antagonism (GW-1100). This finding contrasts sharply with the minimal effects observed with GPR120 antagonism, indicating remarkable receptor selectivity that exceeds simple binding affinity for t10,c12-CLA. This specificity likely reflects distinct binding conformations or allosteric changes induced by subtle structural differences between the receptors—specifically, the positions of double bonds. Recent structural studies of fatty acid-sensing GPCRs have revealed that receptor activation is highly sensitive to ligand geometry [41,42,43], supporting our observation that minor stereochemical variations can involve entirely different signaling pathways. This discrimination at the receptor level provides evidence for how structurally similar bioactive lipids can yield dramatically different cellular responses through selective receptor engagement in mammary cells. Our findings of increased lipid droplet formation with t10c12-CLA treatment contrast with in vivo studies showing reduced milk fat yield [44]. This apparent discrepancy likely reflects: (1) different experimental contexts—isolated cells vs. intact tissue with hormonal regulation, (2) measurement endpoints—intracellular storage vs. secreted lipid, and (3) temporal dynamics—24 h acute treatment vs. chronic dietary supplementation. Importantly, our observation aligns with Harvatine [39] who reported transient lipid accumulation in bovine mammary tissue during early CLA exposure, suggesting a biphasic response. Thus, our findings do not contradict the established anti-adipogenic actions of t10c12-CLA but rather reveal a transient, compensatory increase in lipid storage that may precede or accompany milk fat depression in vivo.

### 4.2. Metabolic Reprogramming: From Synthesis to Storage

Approximately 50% of milk fat originates from plasma-derived fatty acids rather than de novo synthesis [11]. In our model, the accumulation of lipid droplets despite suppressed FASN may reflect: (1) enhanced uptake of CLA isomers themselves or medium lipids via CD36 or FATP transporters, (2) impaired lipolysis due to mitochondrial dysfunction reducing β-oxidation capacity, and (3) a protective response to sequester potentially lipotoxic fatty acids when oxidative metabolism is compromised. Lipidomic analysis would be required to determine whether CLA isomers are directly incorporated into lipid droplets.

The apparent paradox of increased lipid droplet accumulation in the presence of suppressed lipogenic enzymes initially seems contradictory but becomes obvious in terms of cellular energy metabolism. Although earlier investigations relied predominantly on endpoint measurements of enzyme activity or gene expression following CLA treatment [45], we incorporated selective receptor antagonists and functional assays to systematically elucidate the upstream signaling pathways and their metabolic consequences.

The severe mitochondrial dysfunction induced specifically by t10,c12-CLA—evidenced by a 42% reduction in membrane potential and a 65% decrease in the ATP/ADP ratio—may represent a fundamental shift in cellular bioenergetics. When β-oxidation capacity is compromised, cells must store rather than oxidize fatty acids to prevent lipotoxicity [46,47]. This metabolic reprogramming resembles the responses observed in other mitochondrial dysfunction contexts [48], where impaired oxidative metabolism leads to compensatory lipid accumulation as a protective mechanism.

The preservation of mitochondrial function with c9,t11-CLA treatment explains why this isomer can induce increased lipid storage without suppressing lipogenic gene expression. Cells maintain sufficient energy to support both lipid synthesis and storage processes simultaneously. This discrepancy from previous reports may stem from methodological differences, as most prior studies assessed milk fat yield or fatty acid composition in vivo rather than directly visualizing lipid droplet dynamics in isolated mammary epithelial cells [49,50]; thus, this compensatory cellular response may have been missed. For example, some studies have reported antilipogenic effects of c9,t11-CLA in models involving inflammation (e.g., lipopolysaccharide-induced) [9], suggesting context-dependent responses that warrant further exploration in noninflammatory settings such as ours.

### 4.3. PGC-1α Links Receptor Activation to Mitochondrial Dysfunction

The mechanistic pathway linking initial GPR40 engagement to downstream metabolic consequences emerged through our observation that t10,c12-CLA specifically targets PGC-1α, the master regulator of mitochondrial biogenesis. This finding suggests a potential link between receptor activation and phenotypic outcomes, although downstream effectors such as PPARs remain to be investigated.

The 65% reduction in PGC-1α expression observed in our study appears to create a self-perpetuating cycle of decline in mitochondrial activity. PGC-1α, the primary coordinator of mitochondrial gene expression, severely compromises the ability of cells to maintain mitochondrial mass and respiratory capacity [51,52]. This suggests that t10c12-CLA treatment likely leads to a reduction in the number of mitochondria per cell, in addition to impairing the function of existing ones. This mechanism aligns with observations in other metabolically active tissues, where PGC-1α disruption leads to similar bioenergetic crises [53,54].

Importantly, the selective preservation of PGC-1α expression with c9,t11-CLA treatment indicates that mitochondrial disruption is not an inherent consequence of CLA exposure but rather a specific outcome of the interaction of t10,c12-CLA with GPR40. This selectivity underscores the precision of the receptor-mediated recognition system and explains why structurally related compounds can have vastly different cellular effects.

The severe mitochondrial dysfunction induced by t10c12-CLA, evidenced by the reduction in ATP/ADP ratio, decrease in PGC-1α expression, and disrupted mitochondrial morphology, suggests a fundamental impairment in cellular energy metabolism. This degree of ATP depletion likely triggers metabolic reprogramming, as cells must compensate for reduced oxidative phosphorylation capacity. The concurrent suppression of fatty acid oxidation genes (CPT1A, ACADM) indicates that cells lose the ability to utilize fatty acids as an energy source, potentially necessitating increased reliance on glycolysis for ATP generation. This metabolic shift may explain the paradoxical lipid accumulation despite suppressed lipogenic gene expression: fatty acids taken up from the culture medium or released from cellular stores cannot be efficiently oxidized and thus accumulate in lipid droplets. This shift would explain the cell’s ability to survive despite severe mitochondrial impairment, a question raised by our findings.

### 4.4. Transcriptional Reprogramming Confirms Distinct Isomeric Activities

Our transcriptomic analysis provided definitive evidence that, rather than simply differing in potency, CLA isomers engage different cellular programs. The enrichment of lipid metabolism, mitochondrial organization, and stress response genes in the t10,c12-CLA dataset reflects a coordinated response to a cellular crisis. This transcriptional signature extends far beyond simple lipogenic enzyme inhibition, encompassing broad metabolic adaptation, which is consistent with the systemic changes observed in animals experiencing milk fat depression.

The reversal of 78% of these transcriptional changes by GPR40 antagonism provides compelling evidence that this receptor may serve as the primary upstream regulator. The high degree of GPR40 dependence suggests that most of the effects of t10,c12-CLA, both direct and indirect, are ultimately due to this initial receptor interaction.

### 4.5. Implications for Dairy Production and Human Health

Understanding the role of GPR40 as a key mediator suggests potential new avenues for the implementation of more targeted interventions. Our findings provide a mechanistic foundation for precision nutritional strategies in dairy production; for example, GPR40 genotyping could predict animal responsiveness to CLA supplementation, and targeted delivery systems (e.g., rumen-protected formulations with specific isomer ratios) could optimize milk fat modulation while minimizing adverse metabolic effects [55]. Partial GPR40 modulators or combination therapies with mitochondrial protectants could achieve desirable changes in milk composition while preserving cellular health.

Regarding the benefits to human nutrition, our findings provide mechanistic support for the different health effects attributed to different CLA isomers. The minimal metabolic disruption caused by c9,t11-CLA aligns with its generally beneficial profile in human studies [56], whereas the severe cellular effects of the application of t10,c12-CLA may underlie some of the adverse outcomes that have been reported with this isomer [57]. This mechanistic understanding could inform the development of more precise nutritional recommendations and supplemental formulations.

### 4.6. Study Limitations and Future Directions

Despite the mechanistic insights provided by this study, several limitations warrant consideration when our findings are interpreted. First, we conducted our experiments exclusively in primary GMECs cultured in vitro, which, although physiologically relevant, cannot fully represent the complex hormonal and metabolic milieu of the lactating mammary gland in vivo, potentially limiting the direct translation of our receptor-specific findings to whole-animal milk fat depression phenomena.

Similarly, our use of a single CLA concentration (100 μM), which is physiologically relevant for lactating mammary tissue, may not capture the full complexity of the dose–response relationships for receptor activation. The 24 h treatment duration represents acute exposure, whereas dietary CLA supplementation typically involves chronic administration. Long-term studies are needed to determine whether cells develop adaptive responses to chronic t10,c12-CLA exposure or whether mitochondrial dysfunction progresses to irreversible cellular damage.

Although GMECs function well as a physiologically relevant model, species differences in fatty acid metabolism and receptor expression patterns could influence the translational applications of the findings of this study. For example, compared with goats, bovine models, which are more central to dairy production, may exhibit varying levels of GPR40 sensitivity, as suggested by differences in milk fat responses to CLA in cows [7,49]. To address these limitations, we utilized primary cells from lactating animals rather than immortalized cell lines to maintain physiological relevance, and our use of multiple complementary approaches—including functional assays, pharmacological interventions, and transcriptomics—provides convergent evidence supporting our mechanistic conclusions.

Future investigations should prioritize several key experimental approaches. First, the establishment of dose–response curves across physiological CLA concentrations (10–500 μM) would define the therapeutic window for GPR40 activation and potential receptor saturation thresholds. Second, detailed time-course studies spanning 1–14 days would capture the temporal dynamics of receptor activation, potential desensitization, and the evolution of the compensatory lipid accumulation phenotype. Third, validation studies should be extended to bovine and ovine mammary epithelial cells, as species-specific differences in GPR40 expression could influence the applicability of targeted interventions.

Furthermore, although our study definitively links t10,c12-CLA to GPR40-mediated effects, the precise receptor-mediated mechanisms underlying the subtler biological profile of c9,t11-CLA warrant further investigation. Our data demonstrate that GPR120 agonism can robustly suppress lipogenic genes; however, the results of the GPR120 antagonism experiments in this study did not clearly establish GPR120 as the primary mediator of the observed effects of c9,t11-CLA, suggesting that the actions of c9,t11-CLA might be more complex, potentially involving other fatty acid receptors, nonreceptor-mediated pathways, or a combination of subtle effects not fully captured by our current antagonist approach. Hypotheses for future testing include partial GPR120 involvement in inflammatory contexts or the activation of alternative pathways, such as PPAR signaling, which could explain the milder effects observed in the absence of strong GPR40 dependence. Future studies should focus on thoroughly elucidating the specific receptor engagement and downstream signaling pathways of c9,t11-CLA to fully understand its distinct biological profile and its nuanced role in mammary lipid metabolism.

Mechanistically, future investigations should incorporate CRISPR-mediated GPR40 knockout approaches in mammary epithelial cells to definitively establish the roles of relevant receptors. The intermediate signaling cascades linking GPR40 activation to PGC-1α suppression remain undefined; identifying these intermediates could reveal novel intervention points for preserving mitochondrial function while maintaining other GPR40-mediated effects. Additionally, investigating potential synergistic or antagonistic interactions between CLA isomers could aid in the development of optimal supplementation strategies, as both isomers can be present simultaneously in biological systems. The paradoxical increase in lipid droplet accumulation despite the suppression of de novo lipogenesis indicates a critical knowledge gap regarding the interplay between mitochondrial dysfunction and lipid storage dynamics that warrants systematic investigation of autophagy-mediated lipid droplet turnover, endoplasmic reticulum stress responses, and altered fatty acid trafficking pathways. Ultimately, this work suggests a potential paradigm for receptor-mediated metabolic control in mammary tissue that likely extends to other bioactive lipids and metabolic tissues, suggesting that systematic screening of fatty acid–receptor interactions could reveal additional targetable pathways for modulating milk composition and quality to meet both production efficiency and human health goals.

## 5. Conclusions

This study suggests that the different effects of CLA isomers on mammary lipid metabolism arise from their differential engagement of GPR40 with t10,c12-CLA, specifically activating a signaling cascade that suppresses lipogenesis and severely impairs mitochondrial function. This receptor-specific mechanism provides evidence in mammary epithelial cells for isomer-specific effects on milk fat synthesis and reveals principles that may govern how cells distinguish between structurally similar bioactive lipids. These insights advance our understanding of the biology of CLAs from descriptive observations to mechanistic principles, potentially opening avenues for the precise manipulation of milk composition and highlighting the importance of considering cellular energy metabolism in the development of nutritional interventions. The identification of GPR40 as a potential molecular discriminator between the two isomers not only helps explain decades of phenotypic observations but also provides a rational foundation for developing more sophisticated and targeted approaches to modulating mammary lipid metabolism.

## Data Availability

The raw sequence data reported in this paper have been deposited in the Genome Sequence Archive [58] in the National Genomics Data Center [59], China National Center for Bioinformation Beijing Institute of Genomics, Chinese Academy of Sciences (GSA: CRA028216), which is publicly accessible at https://bigd.big.ac.cn/gsa (accessed on 24 November 2025).

## Abbreviations

The following abbreviations are used in this manuscript

ACACA	Acetyl-CoA Carboxylase Alpha
ADP	Adenosine Diphosphate
ATP	Adenosine Triphosphate
CLA	Conjugated Linoleic Acid
FASN	Fatty Acid Synthase
GMECs	Goat Mammary Epithelial Cells
GPCR	G Protein-Coupled Receptor
GPR120	G Protein-Coupled Receptor 120
GPR40	G Protein-Coupled Receptor 40
PGC-1α	Peroxisome Proliferator-Activated Receptor Gamma Coactivator 1-alpha
PPAR	Peroxisome Proliferator-Activated Receptor
SREBP-1	Sterol Regulatory Element-Binding Protein 1
